# A unique mineralization mode of hypermineralized pleromin in the tooth plate of *Chimaera phantasma* contributes to its microhardness

**DOI:** 10.1038/s41598-020-75545-0

**Published:** 2020-10-29

**Authors:** Mayumi Iijima, Mikio Ishiyama

**Affiliations:** 1 Department of Applied Biological Chemistry, Graduated School of Agricultural and Life Sciences, The University of Tokyo, 1-1-1 Yayoi, Bunkyo-ku, Tokyo, 113-8657 Japan; 2 Department of Histology, The Nippon Dental University School of Life Dentistry at Niigata, 1-8 Hamaura-cho, Chuou-ku, Niigata, 951-8580 Japan

**Keywords:** Zoology, Materials science

## Abstract

Tooth plates of the chimaeroids, holocephalian fishes, are unique dental hard tissues. Unlike the teeth of other animals, the tooth plates are located on the roof of the mouth and in the lower jaw. Their tooth plates consist, to a large extent, of lightly mineralized tissue (osteodentin) and hypermineralized tissue (pleromin). Notably, the mineral phase of pleromin is whitlockite, while that of other animals is apatite. Dietary habits of chimaeroids and wearing features of their tooth plates suggest an extreme hardness of pleromin, but this has never been investigated. We examined the microhardness of the tooth plate of *Chimaera phantasma* and found that pleromin in the biting region was extremely hard, comparable with the hardness of mature tooth enamel, whereas the hardness of immature pleromin was lower than that of bovine dentin. The hardness of osteodentin, on the other hand, was equivalent to that of bovine dentin and almost the same throughout the tooth plate. Immature pleromin was sparsely packed with oval crystals of whitlockite and, as pleromin matures, the space between crystals was filled with small intercrystalline materials. The maturing process of pleromin could partly contribute to its remarkable hardness and have some implications for designing novel biomaterials.

## Introduction

The chimaeroid fishes (Holocephali), which appeared about 420 Myr ago^1^ in the Devonian period, belong to a subclass of cartilaginous fish (Chondrichthyes) and is a sister group of the other subclass, Elasmobranchii. The chimaeroid fishes are distinguished among the chondrichthyans by the possession of a unique dental organ, i.e., three pairs of tooth plates, which are not shed or replaced, but evergrowing appositionally (statodont dentition) from caudal to rostral^2–4^. On the other hand, the dentition of elasmobranchs, as represented by shark, that comprises rows of multiple teeth are shed and replaced throughout life (lyodont dentition). Thus, the dentition of chimaeroids contrasts well with that of the Elasmobranchii. Although each of the six tooth plate of chimaeroid was reported to have developed from an individual tooth plate primordium^5^, embryological studies of chimaeroid tooth plates have shown an implication of a compound nature that represents the fusion of adjustment tooth-forming territories^4^. Furthermore, some early holocephalians, e.g., *Helodus*, was found to have a dentition consisting partially of tooth files, that represents a transitional form between tooth files and tooth plates^6–8^.

Tooth plates of chimaeroids are composed of three types of hard tissues: (1) a lightly mineralized tissue that occupies the bulk of the tooth plate called osteodentin^9^, which is also called trabecular dentin^10^, and sclerotic osteodentin^11^; (2) a hypermineralized tissue, named pleromin^12^ which has been also referred to as Kosmin^13^, tubular dentin^14^, orthotrabeculine^15^, and whitlockin^11^. The distribution and morphology of the hypermineralized tissue are different by taxa^16,17^; and (3) a thin veneer called vitrodentin^13^ or the outer dentin^11^ that overlays the tooth plate and quickly wears out on exposed surfaces^4^. Terms for these hard tissues are sometimes changed by various workers with new data^18^. In our study, we refer to the lightly mineralized bulky tissue as osteodentin, the hypermineralized pads and tritoral pad as vascular pleromin, and hypermineralized rods and ovoid as compact pleromin, following our previous papers^2,19^.

Two types of pleromins develop embedded in osteodentin^12,20^; the vascular pleromin is a single large pad that encloses vascular canals, while the compact pleromin is constituent of a series of small bead-like rods and lacks vascular canals. The differentiation of these pleromins is of developmental interest. Similar to tooth enamel, pleromin is a hypermineralized hard tissue. However, pleromin differs from enamel but is more like bone, because pleromin is formed by mesenchyme-derived cells, not by epithelial-derived cells^2^. Furthermore, unlike enamel or enameloid that covers the surface of dentin, pleromin does not cover the entire biting area.

As Ishiyama et al.^2^ reported previously, the mineral phase of hypermineralized pleromin is whitlockite. Whitlockite is assigned the ideal formula Ca_9_Mg(HPO_4_)(PO_4_)_6_^21^. The hard tissue composed of whitlockite is of primary importance in terms of biomineral, because, to our knowledge, the mineral components of the hard tissue in all other vertebrates belong to apatite-group Ca_5_(PO_4_)_3_(OH,F,CO_3_), whose composition differs by species^22^. Extant lungfish (*Lepidosiren paradoxa* and *Protopterus* sp.) also have tooth plates with a constituent of hypermineralized petrodentin^23–25^. However, the petrodentin in the tooth plates consists of hydroxyapatite^26^. Whitlockite is known as the major constituent of the human dental calculus and is involved in the pathological calcification^27,28^, but it is not a constituent of normal dental hard tissues^22^. Thus, pleromin is currently the only example of whitlockitic hard tissue that forms in physiological conditions. Because of this rare mineral phase of pleromin, a recent study proposed to refer to this hypermineralized tissue as “whitlockin”^11^.

Whitlockite gives an X-ray diffraction (XRD) profile similar to that of β-Ca_3_(PO_4_)_2_ (β-TCP) and Mg-containing β-TCP (β-TCa,MgP). Since both whitlockite and β-TCa,MgP contain Mg and exhibit XRD profiles similar to that of β-TCP, it is difficult to distinguish them based on their XRD profiles and Mg content^29^. Thus, a little ambiguity regarding the mineral phase of pleromin^2^ remained needs to be solved. Since HPO_4_ is a key to assigning the mineral as whitlockite, we examined the presence of HPO_4_ in pleromin, using a microscopic attenuated total reflection Fourier transformed infra-red (ATR FT-IR) analysis.

The wear resistance and dietary habit of the chimaeroids, which involves crushing of shellfish^4,11^, implies that the hypermineralized pleromin is extremely hard. To date, however, there has been no study to determine mechanical properties of tooth plates. Here, we report for the first time the hardness of tooth plates in *Chimaera phantasma* (Holocephali) and discuss the hardness in relation to its microstructure.

## Results

### General morphology of the palatine tooth plate

**Figure 1:**
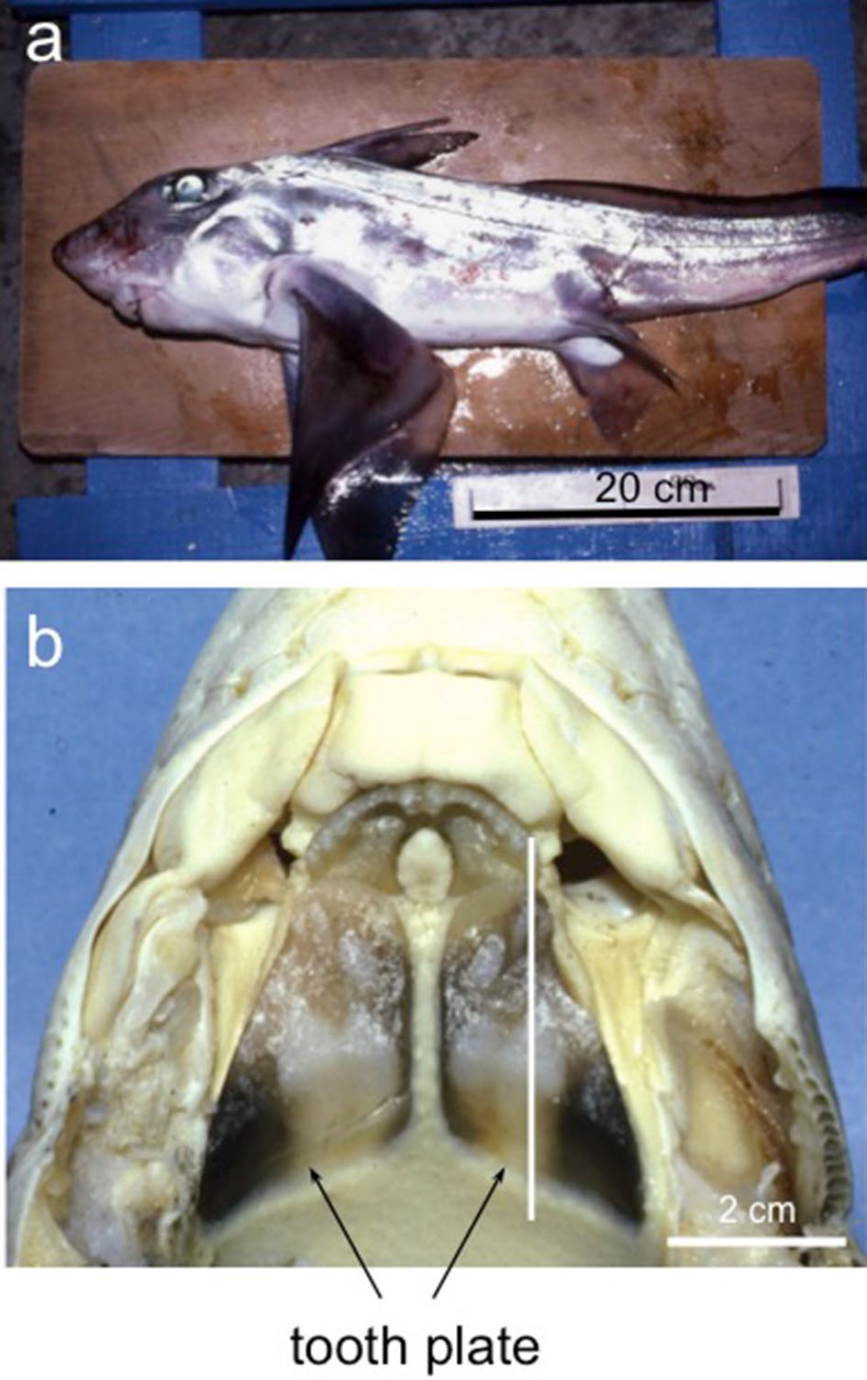
Photographs of the whole body of *Chimaera phantasma* and its tooth plate. (**a**) *Chimaera phantasma* (Shimoda, Japan) used in the present study. (**b**) The upper jaw of *Chimaera phantasma*. A pair of the palatine tooth plates is indicated by arrows. The solid line indicates the direction of cutting.

**Figure 2:**
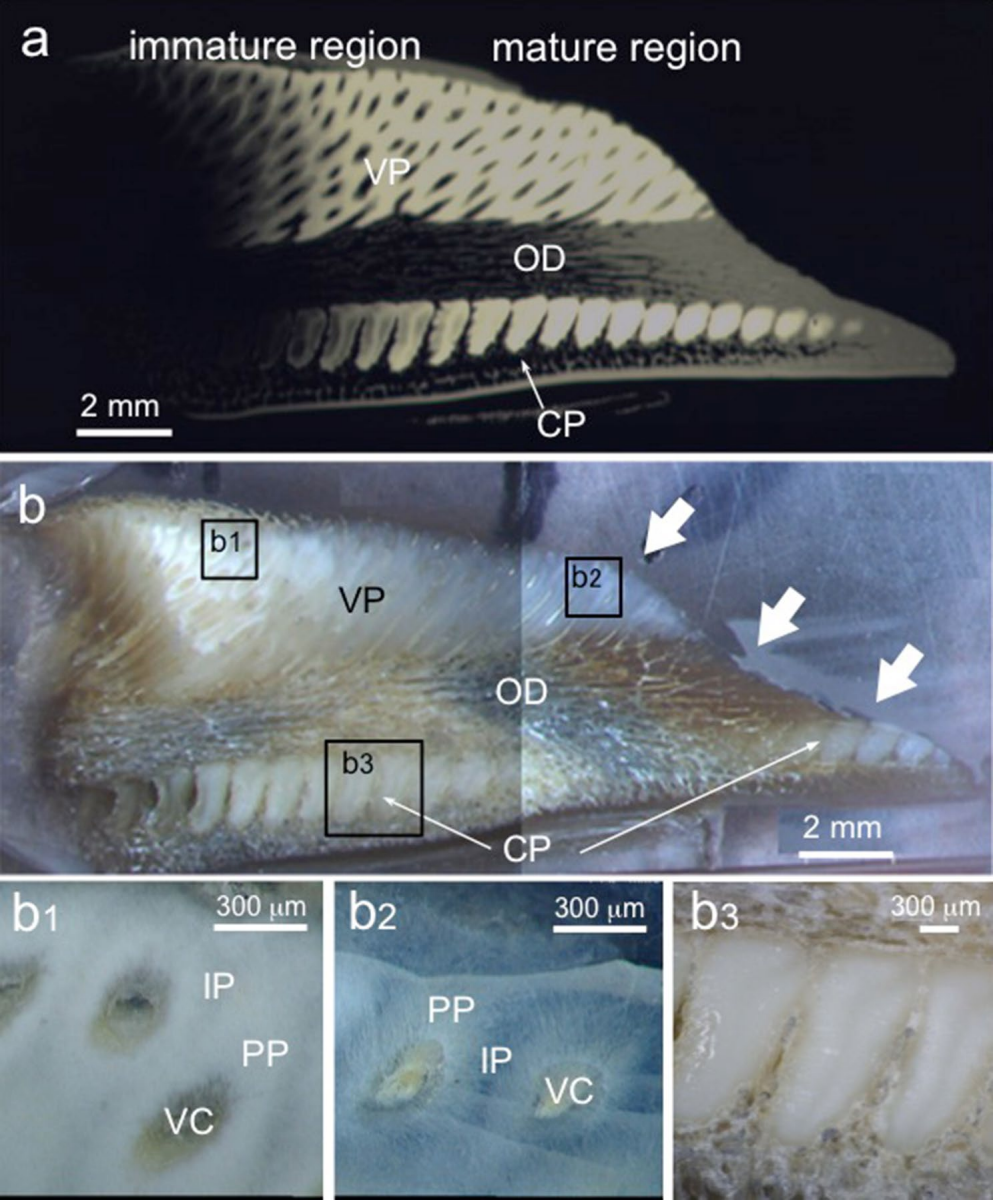
Various images of the longitudinal section of the palatine tooth plate cut along the line in Fig. 1b. (**a**) A contact microradiogram. Radiopaque white regions correspond to hypermineralized pleromin. The upper pleromin is vascular pleromin (VP) and the lower pleromin that forms a row of oval is compact pleromin (CP). Radiolucent gray and black regions correspond to less mineralized osteodentin (OD). Immature pleromin is found in the posterior region (left side), and mature pleromin is present in the anterior region (right side). (**b**) Optical microscopic images. Immature pleromin is opaque and mature pleromin is transparent. Arrows indicate the biting area. Note that pleromin is exposed on the occlusal surface by wear. Enlarged image of (**b1**) immature VP and (**b2**) mature VP, indicated respectively by rectangle (**b1**, **b2**) in (**b**). Pleromin around vascular canals (VC) is peritubular pleromin (PP) and region between PPs is intertubular pleromin (IP). (**b3**) Enlarged image of CP, indicated by rectangle (**b3**) in (**b**).

A contact microradiogram (CMR) (Fig. 2a) and optical microscopic images (Fig. 2b) of a longitudinal section cut along the white line illustrated in Fig. 1 show the distribution of hypermineralized pleromin in the palatine tooth plate. The CMR (Fig. 2a) shows the pleromin as a radiopaque white region and less mineralized osteodentin (OD) (Fig. 2a,b) as radiolucent gray and black regions. The two types of pleromin, vascular pleromin (VP) and compact pleromin (CP), exhibiting different morphologies, are shown in Fig. 2a and b. VP is a large pad of pleromin that contains vascular canals (VC) and occupies an occlusal part of the tooth plate. On the other hand, CP lacks VC and forms rows in the interior region that was named “pearlstrings”^13^. In the posterior region, both VP and CP are radiolucent (Fig. 2a) and opaque (Fig. 2b,b1), indicating that these tissues are immature. As their maturity increases toward the anterior region, pleromin becomes radiopaque (Fig. 2a) and transparent (Fig. 2b,b2). In the biting area (arrows in Fig. 2b), VP is exposed on the occlusal surface by wear. The interior structure of immature and mature VP is shown by Fig. 2b1 and b2, respectively. The enlarged images of VP show that pleromin formed around VC (peritubular pleromin (PP)) is relatively opaque, while pleromin surrounding PP (intertubular pleromin (IP)) is relatively transparent. In contrast, CP is uniformly and densely packed with mineral components (Fig. 2b3).

**Figure 3:**
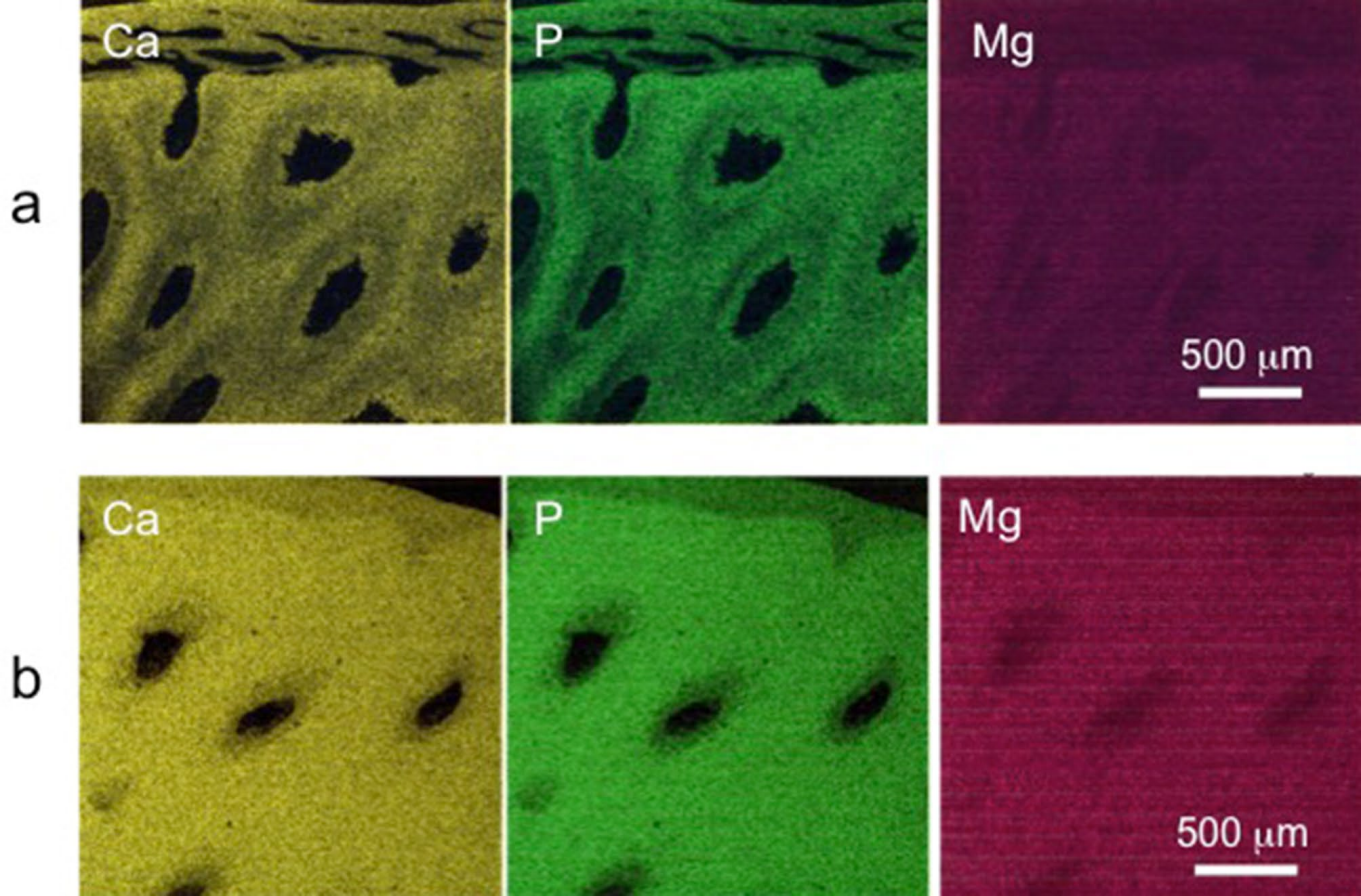
Elemental analysis of vascular pleromin (VP). Distribution maps of Ca, P, and Mg in (**a**) immature VP and (**b**) mature VP. (**a**) and (**b**) correspond, respectively, to areas (**b1**) and (**b2**) in Fig. 2.

Figures 3a and b show the distribution maps of Ca, P, and Mg in the immature and the mature VP, respectively. In the immature VP (Fig. 3a), it is evident that Ca and P localize more in IP than in PP. Whereas in the mature VP, the difference between PP and IP is unclear (Fig. 3b), presumably because of an increase in the calcification level in mature VP (Table 1). In the CP, these ions distributed evenly (Supplementary Fig. S2 online). An energy dispersive X-ray spectrometer (EDS) analysis showed that the Mg content of immature pleromin and mature pleromin was higher than that of osteodentin (Table 1). In VP, the content of Ca, P, and Mg increases as this tissue matures; however, the ratio of Mg substitution for Ca, represented by the Mg/(Mg + Ca) atomic ratio of VP, was approximately 0.044, which indicates that 4.4% of Mg substitutes for Ca in VP, regardless of its maturity. On the other hand, about 1.5% of Mg substitutes for Ca in osteodentin.

**Table 1:** Elemental analysis of vascular pleromin (VP) and osteodentin.

Position	Ca/atm%	P/atm%	Mg/atm%	Mg/(Mg + Ca)
Immature VP-A	4.00 (4)	4.00 (3)	0.18 (1)	0.043
Immature VP-B	8.32 (5)	7.75 (4)	0.39 (1)	0.045
Mature VP	24.6 (1)	22.8 (1)	1.17 (2)	0.045
Osteodentin	3.93 (4)	3.59 (3)	0.06 (1)	0.015

Atomic % of Ca, P, and Mg obtained by EDS analysis and rate of Mg substitution for Ca, Mg/(Mg + Ca) are indicated. The maturity of pleromin increases in the order of immature VP-A< immature VP-B < mature VP. Numbers in parenthesis show 2σ of each data.

### Microhardness of the tooth plate

#### Microhardness of vascular pleromin

**Figure 4:**
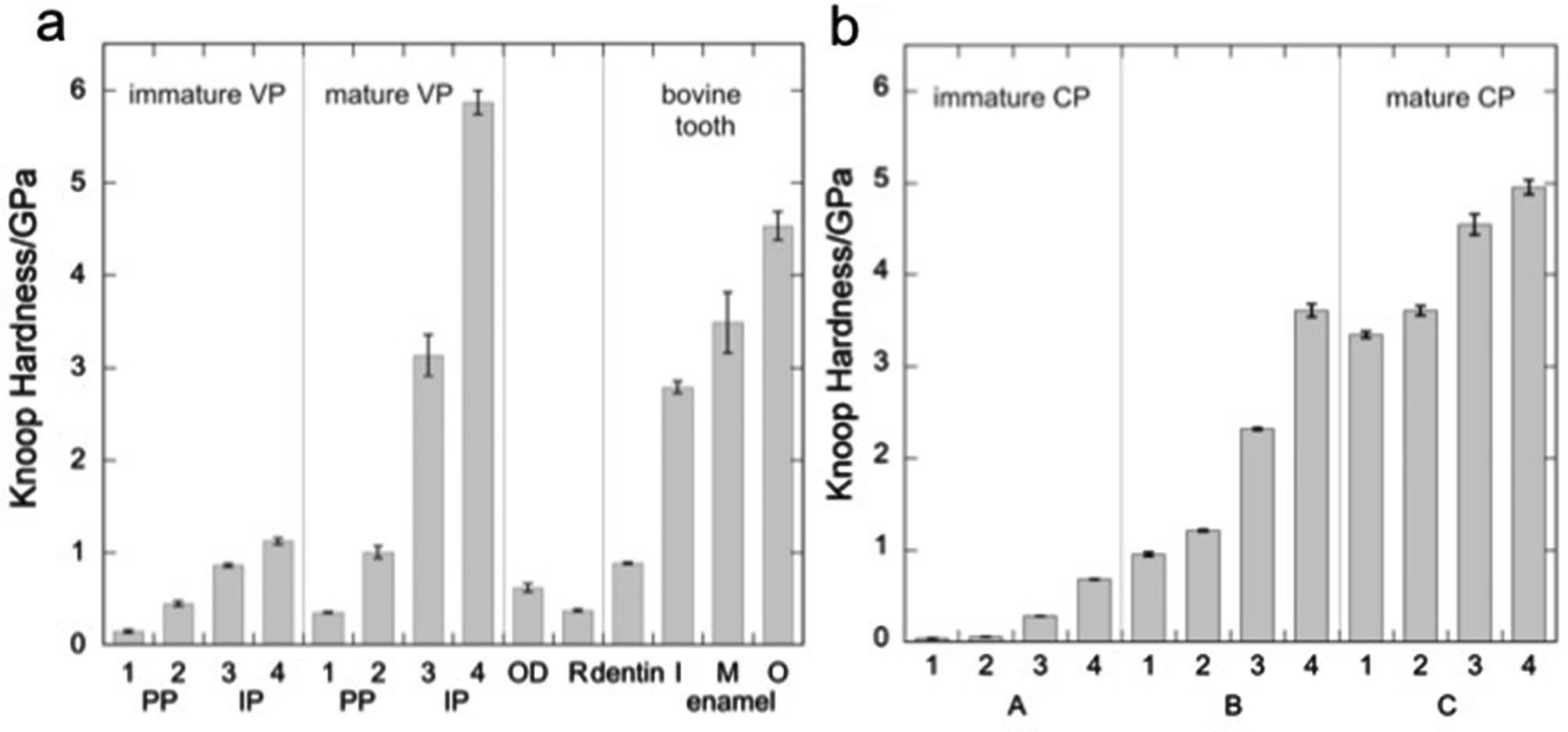
Microhardness of pleromin and other tissues. Knoop hardness (KH) of (**a**) vascular pleromin (VP), osteodentin (OD), and (**b**) compact pleromin (CP) at different maturity. Measured points are indicated in Supplementary Fig. S1 online. In (**a**), KH of resin (R), bovine tooth enamel (inner (I), middle (M), and outer (O) enamel) and dentin are shown for comparison. Error bars show standard deviations for N = 3. Indented points in immature peritubular pleromin (PP) (PP-1, 2) and intertubular pleromin (IP) (IP-3, 4) and those in mature PP (PP-1, 2) and IP (IP-3, 4) are indicated in Supplementary Fig. S1 online. In (**b**), the maturity of compact pleromin (CP) increases in the order of A, B, and C. In each CP, the hardness increases from the outermost region (1 in Supplementary Fig. S1 online) to the central region (4 in Supplementary Fig. S1 online).

The Knoop hardness (KH) of VP in immature and mature regions are shown in Fig. 4a. In both immature and mature VPs, the hardness of IP (IP-3, 4 in Fig. 4a) was higher than that of the PP (PP-1, 2 in Fig. 4a); in the immature VP, the KH of the PP was 0.1–0.5 GPa, whereas the KH of the IP was 0.86–1.1 GPa. The KH increased gradually as the distance from VC increased (Fig. 4a). In the mature VP, the KH of the PP was 0.3–1 GPa (PP-1,2 in Fig. 4a) and that of the IP was 3–5.9 GPa (PP-3,4 in Fig. 4a). The KH increased drastically as the distance from VC increased. The increase of the KH from PP (1 GPa) to IP (3 GPa) in the mature VP was three times higher than that in immature VP (from 0.5 to 0.86 GPa, 1.7 times increase).

In the IP, the hardness increased from the junction of PP-IP toward the center of IP. The maximum KH appeared at the central region of IP, regardless of its maturity. The KH of immature IP (0.86–1.1 GPa, IP-3, 4 in Fig. 4a, Supplementary Fig. S1 online) was as low as that of bovine dentin (0.88 ± 0.01 GPa, Supplementary Table S1 online). In contrast, the KH of central region of IP in the biting region was about 5.86 ± 0.13 GPa (IP-4 of mature prelomin in Fig. 4a, about 600 μm below the surface, Supplementary Fig. S1 online). In two other central regions of IP, the KH was 5.96 ± 0.27 GPa and 5.97 ± 0.13 GPa (both of them located at 300 μm below the surface). These three values show no statistically significant difference (P = 0.900, ANOVA/Tukey) and are larger than the KH of the outer enamel (about 200 µm below the surface) of mature bovine tooth, 4.53 ± 0.14 GPa (Supplementary Table S1 online, Fig. 4a).

#### Microhardness of compact pleromin

The KH of three CPs at different maturity levels (A–C in Supplementary Fig. S1 online) are shown in Fig. 4b. In each CP, the hardness increased from outer regions (1 in Fig. 4b) toward the center (4 in Fig. 4b). The central region was the hardest, regardless of the maturity. In the most mature CP-C, the hardness increased from 2.9 GPa in the outermost region (C-1 in Fig. 4b) to 4.9 GPa in the central region (C-4 in Fig. 4b) (1.7 times increase). In CP-B, the hardness was 0.9 GPa in the outermost region (B-1 in Fig. 4b), while 3.7 GPa in the central region (B-4 in Fig. 4b) (4 times increase). In the most immature CP-A, a large increase was observed, from 39–59 MPa in the outermost region (A-1 in Fig. 4b) to 784 MPa in the central region (A-4 in Fig. 4b; 16 times increase). The CPs that were immature than CP-A were too soft to indent.

#### Microhardness of osteodentin

The KH of OD, 618 ± 43 MPa, that is the average of six different points from posterior to anterior (for indented positions, see Supplementary Fig. S1 online), is indicated in Fig. 4a. The KH of OD was lowest in the most posterior region (point No. 2 in Supplementary Fig. S1 online), 538 ± 5 MPa, while that in the biting region (point No. 6 in Supplementary Fig. S1 online) was relatively high, 659 ± 4 MPa. In the outer OD of the tooth plate covering the CPs (point No. 1 in Supplementary Fig. S1 online), the KH was 624 ± 105 MPa. The KH at point No. 2–No. 6 did not show statistical difference (P > 0.6, ANOVA/Tukey). The average KH of OD was smaller than that of bovine dentin (880 ± 10 MPa) (Fig. 4a).

### Mineral characterization

**Figure 5:**
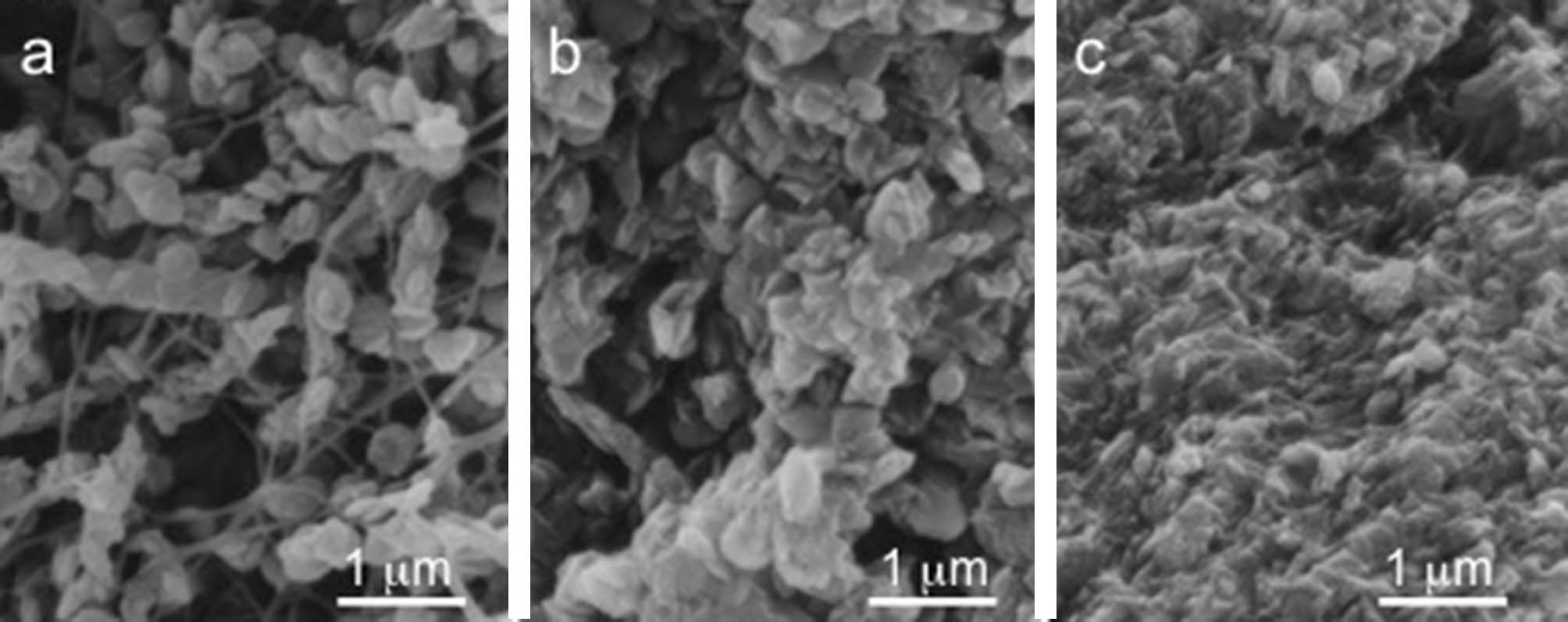
SEM images of pleromin at different maturity. The observed positions are indicated by a, b, and c in Supplementary Fig. S1 online. (**a**) Immature pleromin, (**b**) pleromin under maturation and (**c**) mature pleromin.

The SEM images of VP at different maturity levels are shown in Fig. 5. Three observed positions a, b, and c are indicated in Supplementary Fig. S1 online. Immature pleromin is roughly packed with oval-shaped crystals approximately 300–400 nm in longitudinal direction (Fig. 5a). These crystals are located around fibers and arranged randomly. As pleromin matures, the space between the crystals is filled with small intercrystalline materials (Fig. 5b), and both fibers and oval-shaped crystals become unrecognizable (Fig. 5c). As a result, pleromin grows into a dense tissue. The mineralization process of pleromin undergoes (1) a growth of whitlockite into oval-shaped crystals and (2) a subsequent deposition of intercrystalline materials between the whitlockite crystals.

**Figure 6:**
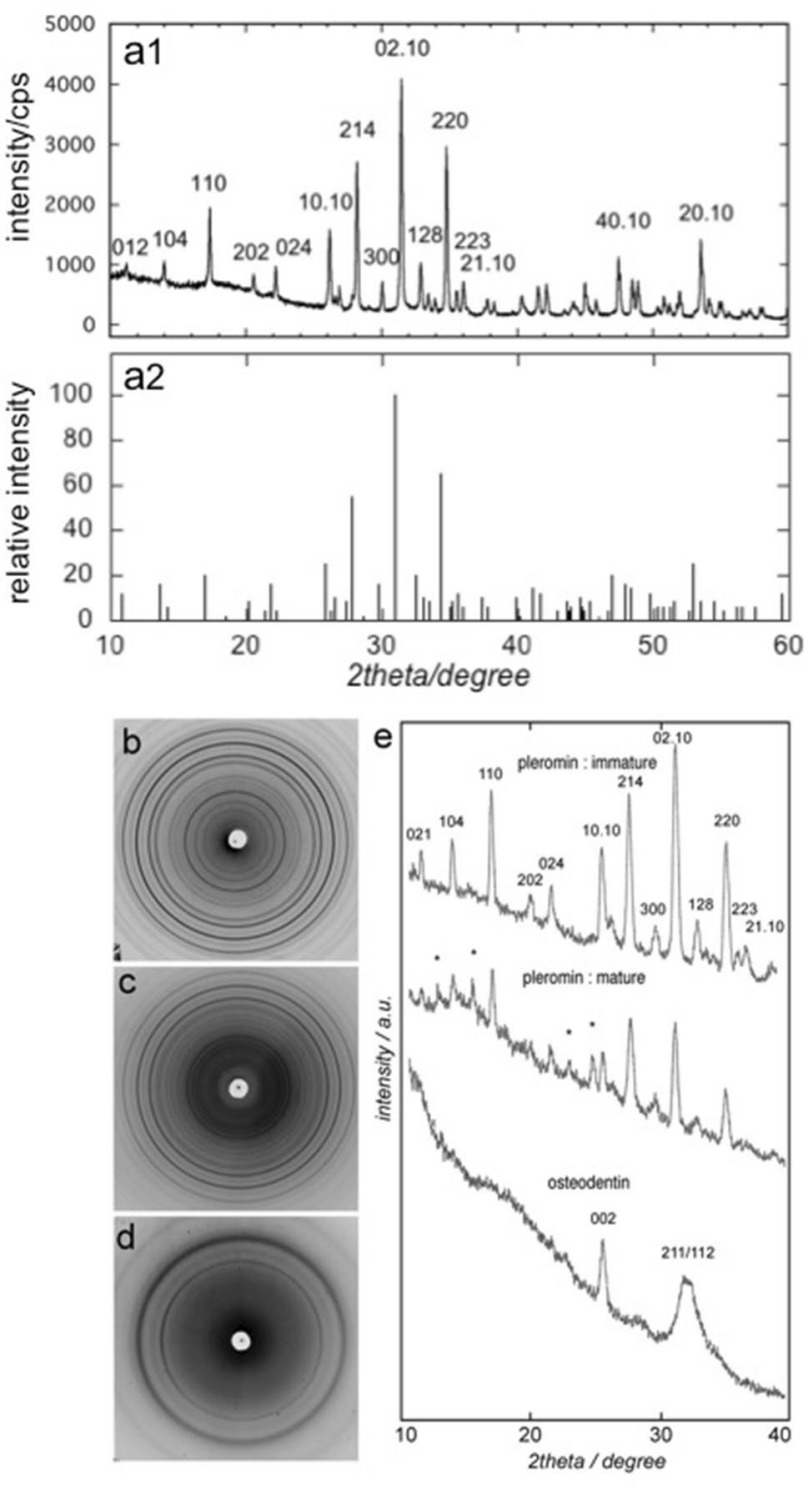
XRD profiles of pleromin and osteodentin. (**a1**) Powder XRD profile of pleromin. (**a2**) XRD peaks for β-TCP (JCPDS 09-0169). Micro-beam X-ray photographs of (**b**) immature pleromin, (**c**) mature pleromin, and (**d**) osteodentin. Note that the Debye–Scherrer rings indicate that crystallites in pleromin (**b**, **c**) and in osteodentin (**d**) have no preferred orientation. (**e**) Densitometric traces of (**b**–**d**). *Indicates peak other than β-TCP.

The powder XRD profile of pleromin is shown in Fig. 6a1, and the peaks for standard β-TCP (JCPDS 09-0169) are indicated in Fig. 6a2. The diffraction peaks of pleromin are extremely sharp, and its profile is similar to the prifile of β-TCP. The diffraction angles (2θ) are, however, slightly higher than those of β-TCP. The refined lattice dimensions are a = 10.408(1) Å and c = 37.244(4) Å. These are smaller than the values reported for β-TCP (a = 10.439(1) Å and c = 37.375(6) Å)^30^. The smaller lattice dimension is consistent with the peak shift to a higher angle.

Figure 6b and c show the micro-beam X-ray photographs of immature and mature pleromin, and Fig. 6e shows their densitometric traces. The XRD peak positions of immature pleromin and those of mature pleromin are equivalent, which indicates that the mineral phase of the immature pleromin is the same as that of mature pleromin. Debye–Scherrer rings in the micro-beam X-ray photographs indicate that the crystallites in pleromin have no preferred orientation, which is consistent with our SEM observation of pleromin (Fig. 5). In mature pleromin, four unidentifiable peaks at 12.5°, 15.3°, 23.5°, and 25.3° in 2 θ (* in Fig. 6e) were observed, while none of these peaks were detected in immature pleromin. On the other hand, our micro-beam X-ray photograph of osteodentin (Fig. 6d) revealed that the mineral component of osteodentin was apatite.

**Figure 7:**
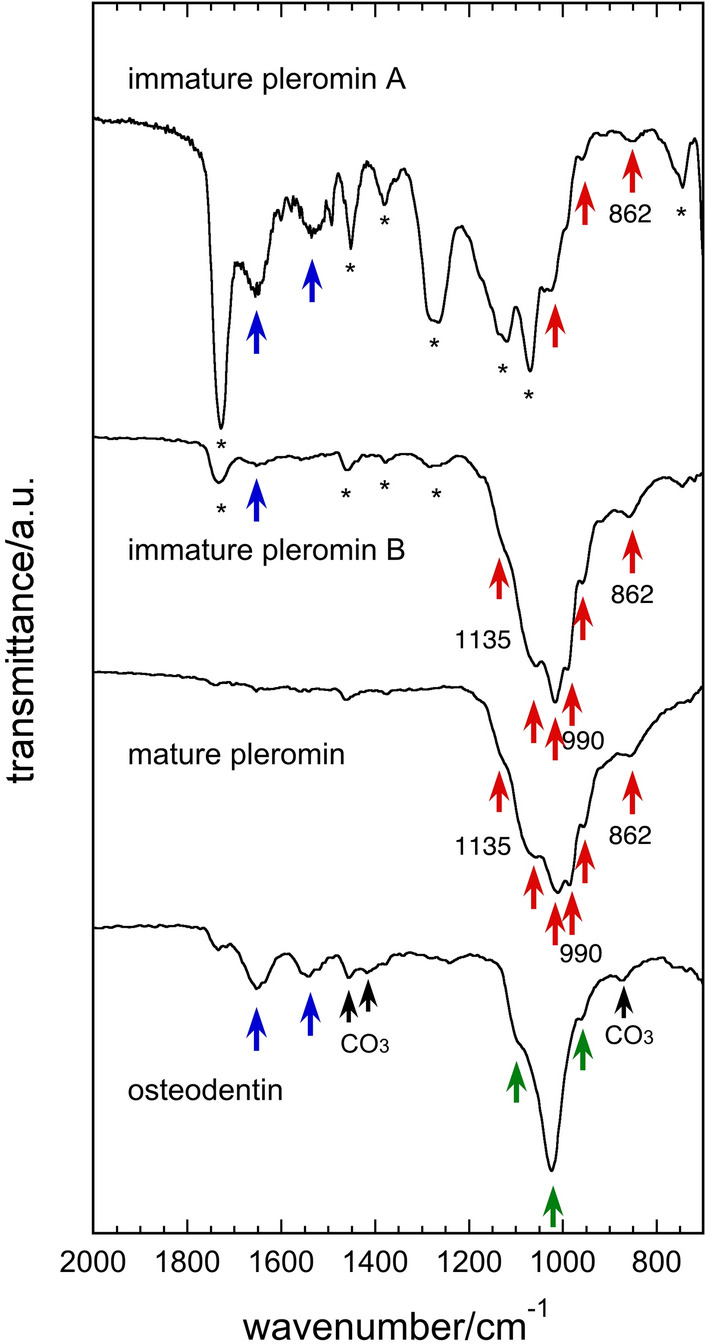
The microscopic ATR FT-IR spectra of pleromin and osteodentin. Immature pleromin with different maturity (A and B), mature pleromin, and osteodentin were analyzed. Bands derived from whitlockite^31,32^ are marked with red arrows. HPO_4_ stretching band at 862 cm^−1^^27^ and υ_3_PO_4_ bands of whitlockite at 990 and 1135 cm^−1^ ^32^ are indicated. In the spectrum of osteodentin, the bands of the PO_4_ groups in the apatite structure are indicated by green arrows. Bands at 1655 cm^−1^ and 1535 cm^−1^ are derived from amide I and amide II^33^ (blue arrows), respectively. Bands at 870, 1418, and 1456 cm^−1^ (black arrows) are derived from CO_3_^–2^ ions in the apatite lattice^34^. Bands marked with * in immature pleromin are derived from the resin.

Figure 7 shows the microscopic ATR FT-IR spectra of pleromin at different maturation levels and osteodentin. Immature pleromin-A is located in the most posterior region, and immature pleromin-B is located between immature pleromin-A and mature pleromin. In immature pleromin-B and in the mature pleromin, bands derived from phosphatic components are prominent. The major absorption bands in the range of 800–1200 cm^−1^ for both immature pleromin-B and mature pleromin overlap with bands derived from whitlockite^31,32^ (red arrows in Fig. 7). The band at 862 cm^−1^ is assigned to HPO_4_ stretching^27^ and the bands at 990 and 1135 cm^−1^ ^32^ are assigned to υ_3_PO_4_ (Fig. 7). It is notable that the band that can be ascribed to whitlockite is also observed in immature pleromin-A, because this suggests that the mineral phase in the early stage is whitlockite. In the spectrum of immature pleromin-A, amide I (1655 cm^−1^) and amide II (1535 cm^−1^)^33^, representatives of protein conformation, are prominent (blue arrows in Fig. 7). We ascribe other bands in the range of 1100–1800 cm^−1^ to the resin (* in Fig. 7, Supplementary Fig. S3 online).

The spectrum of osteodentin is similar to that of apatite, i.e., that of bovine dentin (Supplementary Fig. S4 online). The absorption bands specific to PO_4_ of apatite are indicated in Fig. 7. The bands at 875, 1418, and 1456 cm^−1^ are ascribed to CO_3_^2−^ ions in apatite lattice^34^. These bands are also observed in the ATR FT-IR spectra of bovine tooth enamel (Supplementary Fig. S4 online). Thus, the mineral phase of osteodentin was identified as CO_3_-containing apatite. The bands of amide I (1535 cm^−1^) and amide II (1655 cm^−1^) are also observed in the spectrum of osteodentin (blue arrows in Fig. 7).

## Discussion

Our measurement of the microhardness of the tooth plate of *Chimaera phantasma* revealed a complex hardness distribution in the tooth plate. Although overall hardness of pleromin in the immature region was lower than or equivalent to that of osteodentin, the hardness increased from posterior to anterior regions. It is notable that pleromin in the functioning areas is the hardest of all, approximately 6 GPa. The KH of pleromin was larger than that of the outer enamel (about 200 µm below the surface) of the mature bovine tooth measured in this study (Fig. 4a) and that of the tooth enamel studied previously (2.6–4.3 GPa)^35–38^. In contrast, the overall hardness of osteodentin was approximately 600 MPa. The hardness of pleromin and osteodentin well explains the observation that pleromin and osteodentin show different wear rates, and pleromin becomes exposed on the biting surface upon wear^4,11^. The KH of VP in a posterior immature region was slightly lower than that of osteodentin, probably due to the sparse packing of crystals in this region (Fig. 5a). In the case of CP, immature four CPs younger than CP-A (Supplementary Fig. S1, online) were too soft to indent. This could be ascribed to the empty structure of the earliest CP^11^ (ovoid in^11^). In CP, the KH increased from margin to the center regardless of the maturity of CP (Fig. 4b). This hardness change is consistent with the mineralization process observed by Smith et al.^11^. That is, as the mineralization proceeds, the empty center was filled with granules, indicating that mineralization began in the center of CP^11^. The central part of immature CP contained crystal bundles and was dense, while mineralized periphery of CP was composed with network of tubules, which contained small mineral granules within^11^. This very early stage mineralization pattern could be taken over in the later stage, because the distribution pattern of the microhardness in more matured CP was similar to that of immature CP (Fig. 4b).

Nanoindentation study of tooth enamel^39^ showed that the hardest region (~ 6 GPa) is located within 100 µm of the occlusal surface, and the hardness decreases in regions deeper from the surface and closer to the enamel-dentin junction. This tendency was also observed in bovine tooth enamel examined in this study (Fig. 4a and Supplementary Table S2 online). It is reported that the hardness of tooth enamel shows only a weak correlation with the degree of the prism alignment, but a strong correlation with the chemical composition^39^.

In sea urchin, the microhardness of teeth increases from peripheral regions to the center, which is correlated with an increase in the Mg content of calcite^40^. The maximum hardness of the working zone was reported to be 3.54 GPa, which is much harder than the hardness of other calcitic materials (1.5 GPa for pure calcite)^41^. In the tooth plate of *Chimaera phantasma*, hypermineralized pleromin is composed of Mg-containing whitlockite and pleromin drastically increases in KH with maturation (Fig. 4), as the content of Ca, P, and Mg increases. In fact, the Mg content of mature pleromin was significantly higher than that of the immature pleromin. However, the Mg/(Mg + Ca) ratio remains the same in immature pleromin and mature pleromin (Table 1), indicating that the Mg content of whitlockite in pleromin was largely unchanged during the maturation process of pleromin. This is also supported by our XRD analysis, which shows that the individual peak position of the immature and mature pleromin was unchanged (Fig. 6). Similar relationship between the Mg content and hardness of the hard tissue can be seen in the immature and mature tooth enamel.

The chemical formula of porcine tooth enamel was determined using immature and erupted tooth^42^. That is, Ca_4.155_Mg_0.034_Na_0.191_K_0.004_(HPO_4_)_0.35_(CO_3_)_0.357_(PO_4_)_2.384_(OH)_0.007_ for immature enamel and Ca_4.568_Mg_0.032_Na_0.11_K_0.002_(HPO_4_)_0.143_(CO_3_)_0.256_(PO_4_)_2.712_(OH)_0.378_ for erupted one. According to the chemical formula, 0.8% and 0.7% Mg substitute for Ca in immature and erupted enamel, respectively. These values are much lower than the acceptable quantity of Mg in synthesized Mg-substituted hydroxyapatite (2.1 wt%)^43^. It should be noted that although the erupted tooth enamel is much harder than immature enamel, Mg content was almost the same between them. Therefore, hardening of these hypermineralized tissues can be attributed not principally to Mg, but to some other factors.

In the early stage of pleromin formation, initial crystals deposited in tubular saccules, which is the major constituents of the immature pleromin matrix^19^. The mineral deposition in tubular matrix is also detected by optic observation^11^. These deposits grew into oval-shaped crystals (Fig. 5a). In this stage, the KH of pleromin was much lower than that of osteodentin and bovine dentin (Fig. 4a). In the later maturation stage of pleromin, the space between the oval-shaped crystals was filled with small intercrystalline materials (Fig. 5b,c), majority of which are identical with the oval crysrals in terms of mineral phase (Fig. 6) and composition (Table 1). It should be noted that in the very early mineralization stage of CP (ovoid in^11^), existence of a labile non-crystalline mineral of high Mg content was observed using different optical methods^11^. Furthermore, the non-crystalline mineral transformed rapidly into hypermineralized pleromin^11^ (HD in^11^). In the process of the mineralization, intercrystalline space was filled with irregular shaped mineral deposits (Fig. 5b,c). If the formation of whitlockite starts with Mg-containing amorphous calcium phosphate (Mg-ACP), these deposits could be Mg-ACP, the intermediate transition material from Mg-ACP to whitlockite, and/or whitlockite. It is plausible that unidentified XRD peaks in Fig. 6e were caused by such an intermediate phase. In any case, regardless of the type of mineral phase of the secondary precipitate, an increment of the pleromin density by the secondary precipitation of the intercrystalline materials is a most plausible factor to harden pleromin, rather than the presence of Mg in the mineral. Generally, the space-filling method is effective to make the powdery substance dense. Pleromin could acquire a remarkable hardness through this mineralization mode. This process is distinct from the formation of tooth enamel. In the maturation stage of mammalian tooth enamel formation, the space between the thin ribbon-like immature crystals is filled by the lateral growth of the mother crystals. Enamel crystals thus increase the width and thickness until the crystals are finally in contact with each other^44^.

Extant lungfish also use tooth plates to crush hard pray. The crushing action is reported to occur between powerful jaws, equipped with palatal and lingual tooth plates, and has been observed in the three genera^45^. The tooth plates are composed of osteodentin and petrodentin^23–25^. Petrodentin is formed as a special and unique hypermineralized portion^11,45^. The crushing action implies that the petrodentin is very hard. This has been proved by the hardness measurement. The Vickers hardness of osteodentin is reported to be ~ 430 MPa and that of petrodentin is ~ 3.12 GPa in the plan along the long axis and ~ 2.48 GPa in the plan transverse to the long axis of apatite crystals^37^. Thus, the hardness of the hypermineralized petrodentin was almost comparable to the reported hardness of tooth enamel and shown to be sufficiently high to take the crushing action. In the case of the pleromin of *Chimaera phantasma*, it was not possible to measure the microhardness perpendicular to the biting surface due to its surface condition (see Materials and Methods). However, in contrast to the anisotropic petrodentin, which is composed of the needle shaped hydroxyapatite crystals^26^, pleromin is supposed to be more isotropic than petrodentin: Pleromin is composed of oval-shaped and irregular shaped fine whitlockite crystals without preferred orientation (Fig. 5). Micro-beam XRD profiles (Fig. 6e) indicate that there is no preferred orientation in pleromin constituting crystals. Therefore, mechanical property is supposed to be relatively isotropic. Although chimaera and lungfish belong to different class and the mineral phase of the hypermineralized tissue in the tooth plate is different, both tooth plates have a common nature; that is, a hard and hypermineralized tissue develops in a soft and less mineralized matrix. There might be a common mechanism to form hypermineralized tissue in the osteodentin matrix between them.

The uniqueness of pleromin in *Chimaera phantasma* may be highlighted by comparison with the dental tissues of other fishes. Sharks (Elasmobranchii) also belong to cartilaginous fish (Chondrichthyes) and are the closest relative of *Chimaera*. Nevertheless, the dental tissues of sharks differ considerably from those of *Chimaera*. Notably, sharks have a series of teeth on the jaws, and the hypermineralized part of teeth (enameloid) entirely covers dentin. Furthermore, the mineral phase of shark enameloid is fluorapatite. Unlike sharks, lungfish have tooth plates, but the hypermineralized tissue in tooth plates (petrodentin) is composed of hydroxyapatite^26^. Thus, chimaeroid pleromin is distinct among biominerals in vertebrates, which are composed of calcium phosphates. Our findings of the relationship between the mineralization mode and the hardness would help creating novel biomaterials.

## Conclusion

The hypermineralized portion of the tooth plate of *Chimaera phantasma* is composed of whitlockite that shows a remarkable hardness, comparable to the hardness of tooth enamel. The hardness of immature pleromin increases strikingly, as it grows toward its functioning region, and as it undergoes hypermineralization. The mineral phase and the rate of Mg substitution for Ca of the mature prelomin are equivarent to those of the immature pleromin. We hypothesize that a striking increase in hardness could be correlated with the mineralization process of pleromin, which involves (1) the growth of whitlockite into oval-shaped crystals, and (2) the subsequent deposition of intercrystalline materials between the whitlockite crystals. A drastic increment of the hardness of pleromin in the biting region of the tooth plate should be the key for chimaerids to preying upon hard-bodied diet.

## Methods

### Sample preparation

Tooth plates of *Chimaera phantasma* (adult, length ~ 80 cm, Shimoda, Japan) (Fig. 1a) was used. Palatine tooth plates (arrowed in Fig. 1b) was removed from the upper jaw and subsequently soaked in 10% formalin with neutral pH for 48 h, dehydrated by immersing the tooth plate in 10–100% ethanol progressively for 60 min in each concentration, and embedded in polyester resin (Rigolac). The resin block was cut longitudinally along the line in Fig. 1b into two half with a diamond blade (Buehler Ltd.) at a low speed. One of the halves of the resin block was used for the measurement of microhardness. The cut surface was polished with waterproof SiC-abrasive papers (#1000–#2000) and Al_2_O_3_-polishing papers with Al_2_O_3_ particle size of 3 μm (#4000), 1 μm (#8000), and finally 0.3 μm (#15000). The polished block was cleaned using an ultrasonic cleaning apparatus in distilled water for 3 s.

The other half of the resin block was cut into longitudinal cross sections (thickness of ~ 200 µm), using a saw microtome (Leitz-1600) and used for characterization of the mineral phase of pleromin and osteodentin. Since the biting surface of the tooth plates is uneven and have lots of concavities, it was not possible to make a mirror polishing surface for hardness measurement. Therefore, hardness measurement was conducted normal to the biting direction using the longitudinally cut surface.

### Observation of general morphology of the tooth plate

To determin the position to analyze, the morphology and distribution of hypermineralized pleromin in the longitudinal cross section was examined using an optical microscope and a contact microradiography (CMR) (Sofron, SRO-M50, 4 kV, 4 mA). CMR was performed using a polished section with a thickness about 70 µm.

### Measurement of microhardness

The Knoop hardness (KH) was measured for (1) immature vascular pleromin (VP) in posterior regions and mature VP in anterior regions (b1 and b2 in Supplementary Fig. S1 online), (2) three compact pleromin (CP) with different maturity levels (A, B, and C in Supplementary Fig. S1 online), and (3) six points in osteodentin (OD) (1–6 in Supplementary Fig. S1 online), using a Knoop microindentor (Shimadzu, Microindentor HMV-2000). Indentation of VP was conducted in arrays directed from an inner part of the PP towards the center of the IP (Supplementary Fig. S1 online). Three indented CPs are indicated in Supplementary Fig. S1 online as A, B, and C. Maturity of the CPs increases in the order of A, B, and C. In each CP, four points from peripheral regions to the center were indented (Supplementary Fig. S1 online). CPs that are more immature than A, were too soft and could not be indented. Indentation of the OD was performed at six different points in the regions beginning from a posterior region to more anterior regions (Supplementary Fig. S1 online).

Mature VP and CP were indented with a load 200 or 100 gf for 30 s. A small load was applied to soft regions; that is, immature VP and CP in posterior regions and osteodentin, were loaded at 50 or 10 gf for 30 s. Immediately after indentation, length of the long indentation diagonal was measured three times. Knoop hardness number (KHN) (kgf/mm^2^) was obtained for each scale reading. Then, mean KHN and its standard deviation (SD) (N = 3) were calcurated for one indentation point. To represent Knoop hardness (KH), KHN was converted into GPa. For comparison, the hardness of bovine molar tooth, which was embedded and prepared equally, were measurd. For enamel, outer surface, middle regions, and inner regions near the enamel-dentin junction were indented. For dentin, the central region between the enamel-dentin junction and dental pulp was indented. The KH of enamel and dentin of a bovine tooth were obtained by measureing the KHN of three closely spaced positions. The Knoop microindentor was adjusted using a standard stainless steel block with hardness of 9.4–10.2 GPa before measurement of the sample. The standard stainless steel block gave KH of 10.20 ± 0.02 GPa. All the measurements were performed at room temperature.

Multiple comparison tests of the mean hardness of various points were performed using KaleidaGraph (ver. 4.0). The data were statistically analyzed by a one-way classification analysis of variance (ANOVA) with the Tukey posterior multiple comparison.

### Characterization of the mineral phase

To identify the mineral phase in a specific position of pleromin and osteodentin, a micro-Laue camera (Rigaku) was used. Longitudinal cross sections of a tooth plate (Fig. 2b) were fixed onto a sample-holder, and a selected area of immature and mature vascular pleromin (a, c in Supplementary Fig. S1 online) and a central part of osteodentin (2 in Supplementary Fig. S1 online) were irradiated with a focused X-ray beam using a collimator of 100 µm diameter. A micro-Laue photograph of each position was taken with an exposure time of 20 h (56 kV, 200 mA, CuKα). The lattice dimension of whitlockite was refined by means of a powder X-ray diffractometer (Rigaku, Rint2500, 56 kV, 200 mA, CuKα) using 20 peaks. Diffraction angle (2θ) of each peak was corrected using Si powders.

To analyze a specific position of pleromin and osteodentin, microscopic Fourier Transformed Infra-red (FT-IR) analysis was performed using the attenuated total reflection (ATR) method (Shimadzu, FT-IR 8800). For measurement, a longitudinally sectioned tooth plate (Fig. 2b) was used. Three positions of VP with different maturity levels (a, b, c in Supplementary Fig. S1 online) and a central portion of osteodentin (3 in Supplementary Fig. S1 online) were examined. Resin that surround sample was also measured (Supplementary Fig. S3 online). Each point was lightly pressed with a Ge-prism and an IR spectrum from a small area (100 × 100 µm^2^) was obtained. For comparison, the spectra of bovine tooth enamel and dentin were obtained using a thin section of the tooth and the same ATR FT-IR instrument (Supplementary Fig. S4 online).

After a longitudinal cross section was coated with a thin layer of carbon using a carbon evaporator, the tooth plate was observed by a scanning electron microscope (SEM) (HITACHI, S4500) and atomic analysis of the same area was conducted by an energy dispersive X-ray spectrometer (EDS) (HORIBA, EMAX-7000), equipped with SEM. The content and distribution of calcium (Ca), phosphorus (P), and magnesium (Mg) of these areas were analysed under a 20 kV of operating voltage, 0.44 nA of probe current, and aquisition time of 3000 s. Three positions of vascular pleromin (VP) at different maturity levels (a–c in Supplementary Fig. S1 online) were examined.

### Ethical approval

All methods were carried out in accordance with relevant guidelines and regulations.

All experimental protocols were approved by a named institutional and/or licensing committee.

This study was approved by Animal Experiment Committee of the Nippon Dental University School of Life Dentistry at Niigata.

## Supplementary information

Supplementary Information.
