# Supplementary material for: A unique mineralization mode of hypermineralized pleromin in the tooth plate of Chimaera phantasma contributes to its microhardness
Source: Sci Rep. 2020 Oct 29;10:18591. doi: 10.1038/s41598-020-75545-0 (PMC7596707; doi:10.1038/s41598-020-75545-0)
Supplement: Supplementary file 1 — Supplementary Information. [file 41598_2020_75545_MOESM1_ESM.pdf]

Supplementary Information

A Unique Mineralization Mode of Pleromin in Tooth Plate of *Chimaera phantasma* Contributes to Its Microhardness

M.Iijima[1]* & M.Ishiyama[2]

* corresponding author

Affiliation:

1 Department of Applied Biological Chemistry, Graduated School of Agricultural and Life Sciences, The University of Tokyo, 1-1-1 Yayoi, Bunkyo-ku, Tokyo 113-8657, Japan
2 Department of Histology, The Nippon Dental University School of Life Dentistry at Niigata, 1-8 Hamaura-cho, Chuou-ku, Niigata 951-8580, Japan

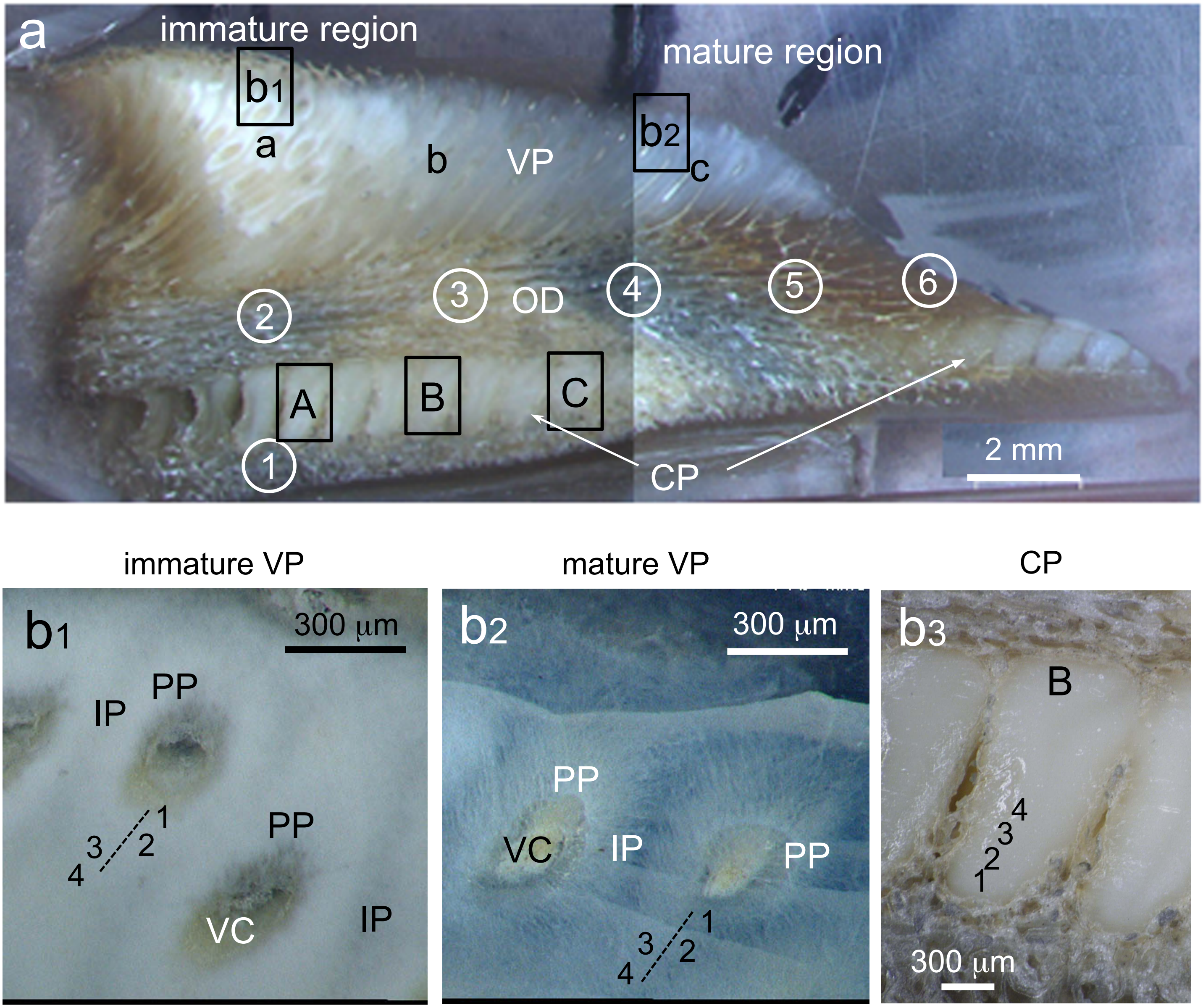

Supplementary Figure S1 (a) An optical microscopic images of the longitudinal section of the palatine tooth plate cut along the line in Figure 1b. Analyzed positions for Knoop Hardness (KH) : (1) (b1) immature vascular pleromin (VP) and (b2) mature VP, in arrays directed from the inner part of the peritubular pleromin (PP) towards the center of the intertubular pleromin (IP), (2) three compact pleromin (CP) with different maturity marked with A, B, and C, and (3)six position of osteodentin (OD) marked with 1–6. Indented positions, 1, 2, 3, and 4, are indicated in b1, b2, and b3. For SEM, FT-IR, and micro-beam XRD: a, b, and c.

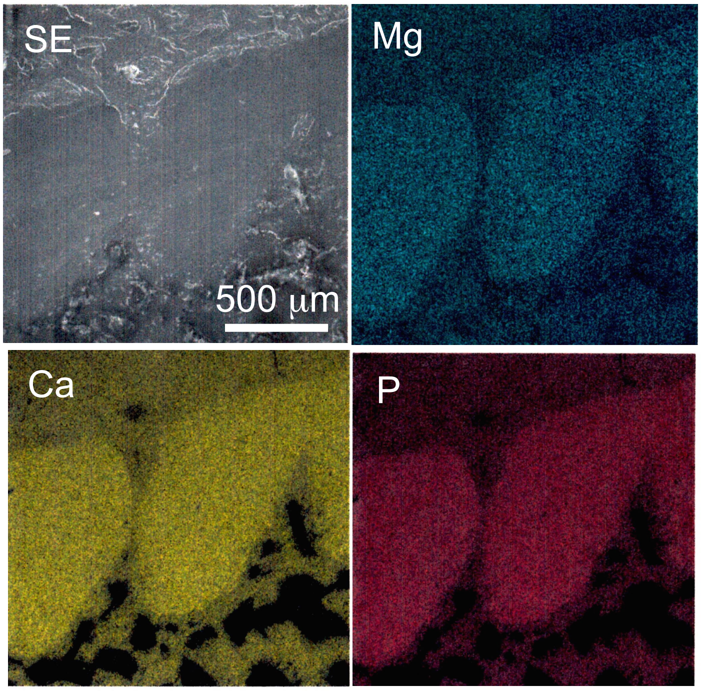

Supplementary Figure S2 SE image and distribution map of Ca, P, and Mg in the mature compact pleromin.

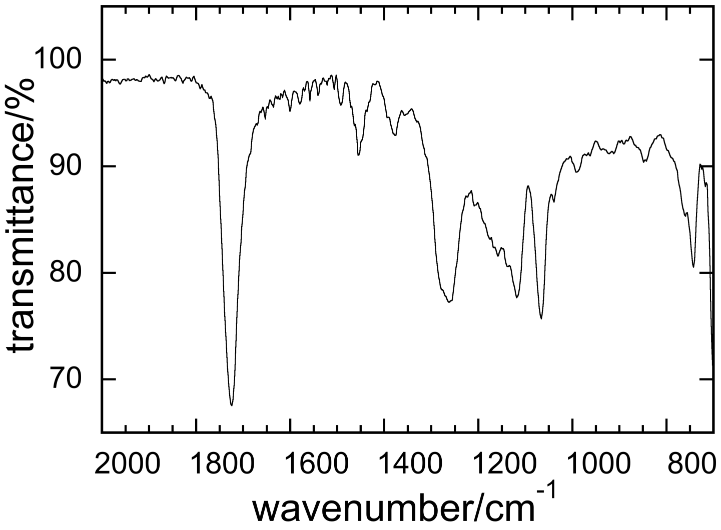

Supplementary Figure S3 ATR FT-IR spectrum of the resin used to embed sample.

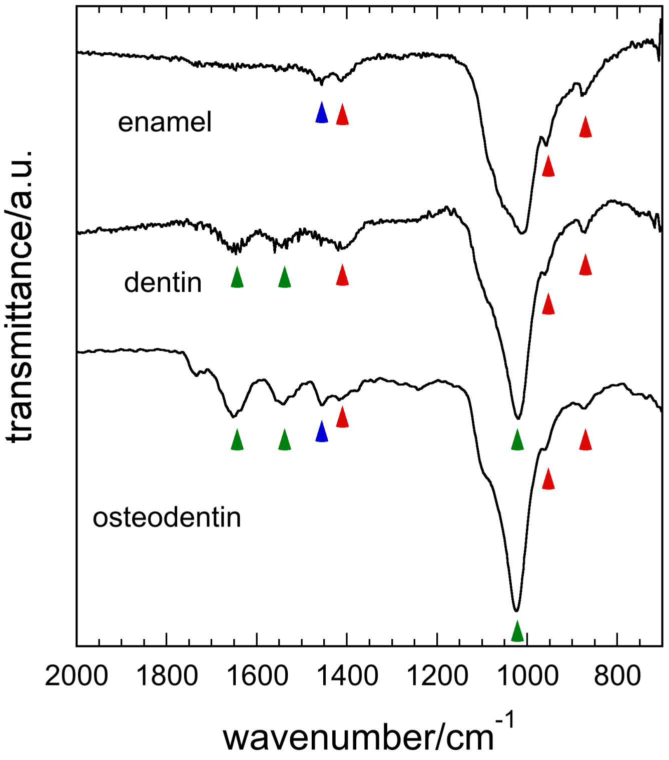

Supplementary Figure S4 ATR FT-IR spectra of the bovine tooth enamel and dentin, and osteodentin. Bands of mutual wavenumber with osteodentin and bovine dentin are marked with green-arrow head, that with osteodentin and enamel with blue one, and that with enamel, dentin, and osteodentin with red one.

Supplementary Table S1 Knoop Hardness (KH) of bovine dentin and enamel, resin, and standard steel. N=3

position	KH (GPa)
dentin	0.88±0.01
inner enamel	2.79±0.06
middle enamel	3.49±0.33
outer enamel	4.53±0.15
resin	0.37±0.01
standard steel	10.19±0.17

Supplementary Table S2 Knoop Hardness (MPa) of six points of the osteodentin. Measured points are indicated in Supplementary Figure S1. In each measurement, N=3. Average±SD for 6 positions are 618±43.

position	KH (MPa)
1	624±105
2	538±5
3	615±9
4	627±46
5	648±22
6	659±4
